# Nursing Team Composition and Mortality Following Acute Hospital Admission

**DOI:** 10.1001/jamanetworkopen.2024.28769

**Published:** 2024-08-19

**Authors:** Peter Griffiths, Christina Saville, Jane Ball, David Culliford, Jeremy Jones, Francesca Lambert, Paul Meredith, Bruna Rubbo, Lesley Turner, Chiara Dall’ora

**Affiliations:** 1 School of Health Sciences, University of Southampton, Southampton, United Kingdom; 2 Portsmouth Hospitals University Trust, Portsmouth, United Kingdom; 3 National Institute for Health Research Applied Research Collaboration, Wessex, United Kingdom; 4 Statistical Sciences Research Institute, University of Southampton, Southampton, United Kingdom

## Abstract

**Importance:**

Many studies show the adverse consequences of insufficient nurse staffing in hospitals, but safe and effective staffing is unlikely to be just about staff numbers. There are considerable areas of uncertainty, including whether temporary staff can safely make up shortfalls in permanent staff and whether using experienced staff can mitigate the effect of staff shortages.

**Objective:**

To explore the association of the composition of the nursing team with the risk of patient deaths.

**Design, Setting, and Participants:**

This patient-level longitudinal observational study was conducted in 185 wards in 4 acute hospital trusts in England between April 2015 and March 2020. Eligible participants were patients with an overnight stay and nursing staff on adult inpatient wards. Data analysis was conducted from month April 2022 to June 2023.

**Exposure:**

Naturally occurring variation during the first 5 days of hospital admission in exposure to days of low staffing from registered nurses (RNs) and nursing support (NS) staff, the proportion of RNs, proportion of senior staff, and proportion of hospital-employed (bank) and agency temporary staff.

**Main Outcomes and Measures:**

The primary outcome was death within 30 days of admission. Mixed-effect Cox proportional hazards survival models were used.

**Results:**

Data from 626 313 admissions (319 518 aged ≥65 years [51.0%]; 348 464 female [55.6%]) were included. Risk of death was increased when patients were exposed to low staffing from RNs (adjusted hazard ratio [aHR], 1.08; 95% CI 1.07-1.09) and NS staff (aHR, 1.07; 95% CI, 1.06-1.08). A 10% increase in the proportion of temporary RNs was associated with a 2.3% increase in the risk of death, with no difference between agency (aHR, 1.023; 95% CI, 1.01-1.04) and bank staff (aHR, 1.02; 95% CI, 1.01-1.04). A 10% increase in the proportion of agency NS was associated with a 4% increase in risk of death (aHR, 1.04; 95% CI, 1.02-1.06). Evidence on the seniority of staff was mixed. Model coefficients were used to estimate the association of using temporary staff to avoid low staffing and found that risk was reduced but remained elevated compared with baseline.

**Conclusions and Relevance:**

This cohort study found that having senior nurses in the nursing team did not mitigate the adverse outcomes associated with low nurse staffing. These findings indicate that while the benefits of avoiding low staffing may be greater than the harms associated with using temporary staff, particularly for RNs, risk remains elevated if temporary staff are used to fill staffing shortages, which challenges the assumption that temporary staff are a cost-effective long-term solution to maintaining patient safety.

## Key Points

**Question:**

Is variation in the composition of the nursing team on a hospital inpatient unit associated with risk of patient death?

**Findings:**

In this longitudinal cohort study of 626 313 patient admissions in 4 centers, there was a statistically significant increase in the risk of death among patients exposed to days of low nurse staffing or high proportions of temporary staff. When low staffing was averted using temporary staff, the risk was reduced but remained elevated compared with the baseline.

**Meaning:**

These findings suggest that the risk of death associated with low nurse staffing is only partly mitigated by using temporary staff to remedy shortfalls.

## Teaser

This cohort study examines associations of nursing team composition with the risk of death among adult patients with acute hospital admission in England.

## Introduction

Many studies and inquiries into poor care have demonstrated the adverse consequences of inadequate nurse staffing on acute hospital wards, including increased risk of patient death and suboptimal experiences for both patients and staff.^1,2,3^ While existing evidence can be used to make a compelling case for avoiding low staffing and increasing the availability of registered nurses (RNs), safe and effective staffing is not solely determined by the number of staff, but involves many factors including the composition, skill, and experience of the nursing team.^4,5^ Although some consideration has been given to the mix of staff between RNs and nursing support (NS) staff, much less attention has been given to other aspects of the mix of staff.

Studies on nursing skill-mix in acute care hospitals typically define skill-mix as the proportion of RNs among all those delivering hands-on nursing care.^6^ The wider team includes NS staff who work under the supervision of RNs, including nursing assistants, who are not professionally registered, and nurses with lower-level qualifications such as licensed practical nurses and nursing associates, a role recently introduced in the UK to support RNs.^6^ Most findings suggest that a skill-mix with a lower proportion of RNs is associated with more adverse outcomes^6^ and either unchanged or increased net costs.^7^ However, there is ongoing interest in modifying skill-mix to contain costs and alleviate the effects of RN shortages.^8^

Temporary staff are often used to compensate for staff shortages or as a potentially efficient way of responding to variations in demand. Some studies have found a higher risk of adverse events with high levels of temporary staff. Unfamiliarity with the care setting is hypothesized to lead to inefficiency, reduced continuity, and, hence, increased risk to patients.^9,10,11^ However, other studies have found no adverse outcomes from using temporary staff to maintain staffing levels.^12,13^ Recent research highlights differences in the impact of external agency nurses and temporary staff directly employed by hospitals.^14^ The same study also identified that more senior RNs were more productive, thus raising the possibility that more senior staff could partially mitigate the adverse effects of low staffing.^14^ Within England, recent changes have focused on improving the skills of staff who provide support to nurses by introducing a new role, the RN associate,^15^ but no research has thus far explored the effect of staff-mix among support staff.

In this article, we aim to fill the gaps and uncertainties in existing literature by presenting findings from a multisite study in England. We investigated the association of the risk of death following an acute admission with nursing team composition. Specifically, we examined the impact of low staffing, the proportion of RNs, the seniority of both RNs and NS staff, as well as the proportion of temporary staff.

## Methods

This cohort study was approved by the Health Research Authority and the University of Southampton Ethics Committee. The research was conducted in accordance with the Declaration of Helsinki^16^; written informed consent was not required because data was deidentified with no reasonable possibility of identifying any living person. Patients in the National Health Service have the option of opting out of use of their data in this fashion for research purposes. Reporting followed the Strengthening the Reporting of Observational Studies in Epidemiology (STROBE) reporting guideline. We used anonymized patient records and nurse roster data from 4 acute hospital trusts in England from April 2015 to March 2020 to explore longitudinal associations of staffing levels and staff-mix with outcomes. The trusts were diverse in many respects, including size and teaching status (and eTable 1 and eTable 2 in Supplement 1). Eligible patients were those with elective and emergency admissions with an overnight stay in an adult medical, surgical, or intensive care ward.

### Data and Variables

The primary outcome was death from all causes within 30 days of admission (including death after discharge). We used details in the patient record to calculate an estimated risk of death using the published Summary Hospital Mortality Indicator (SHMI) model,^17,18^ based on age, diagnosis (*International Statistical Classification of Diseases and Related Health Problems, Tenth Revision *[*ICD-10*]), method of admission (emergency vs elective) and comorbidities. We applied the April 2019 model, developed from national data for the previous 3 years, which coincided with the study period.

Staffing data consisted of worked shifts from ward rosters. Nursing staff were identified as RN or NS from their pay band, using the national grading system.^19^ Jobs in the English National Health Service are evaluated and allocated into 1 of 9 major bands based on criteria including the levels of judgement, knowledge, and skills required, with band 5 and above used for RNs and 4 or lower for NS staff.^20^ We calculated the number of hours provided by each of these staff groups and divided these by patient-days to calculate hours per patient-day (HPPD) for both RNs and NS staff. We identified temporary staff employed directly by the hospital (ie, bank) or hired through an external agency and calculated the hours worked within each staff group. These data were used to calculate variables to indicate understaffing and the mix of staff for each day of each patient’s stay.

We estimated the expected staffing for both RN and NS staff using the mean staffing levels (in HPPD) for each ward. Where we identified sustained change in the most frequent diagnostic categories, main specialty, sex, modal age group, mix of elective vs emergency admissions, proportion of overnight stays, or patient occupancy, we divided the time series and calculated the expected staffing level for each period. We identified days when a patient experienced staffing below expected, using days below the mean HPPD as the primary threshold, with other thresholds above and below the mean tested in a sensitivity analysis. We focused analysis on exposures that occurred during the first 5 days of the hospital stay, accounting for the majority of the stay for most patients, although we assessed the sensitivity of results using longer and shorter exposure periods.

We identified the daily proportion of hours worked by RNs and considered the mix of grades within the RN and NS staff groups as a proxy for experience and seniority. We calculated the percentage of senior RNs based on proportion of RN hours delivered by band 6 and above. Band 5 is used for the majority of staff nurses, with band 6 and above used for senior staff nurses, ward managers, charge nurses, and their deputies.

We calculated the percentage of senior NS staff based on the proportion of NS hours delivered by band 4 staff. Whereas there are no formal training requirements for NS staff as a whole, band 4 is generally used for those with extensive formal training and experience including RN associates, a relatively new role with apprenticeship training leading to a foundation degree.^15^ We calculated the percentage of RN hours from temporary staff, separating hours staff employed through the hospital’s own bank and from an external agency. Similarly, we calculated the percentage of temporary NS hours.

We linked staffing levels and the mix of staff for each day of each patient’s stay to the patient records. Patient-days not matched to RN staffing were treated as missing, although patient-days where NS hours were 0 were retained because wards can operate without support staff. The previous day’s staffing values were carried forward in the case of missing values.

### Statistical Analysis

We used mixed-effect Cox proportional hazards survival models to handle time-varying covariates because patients experience daily variations in staffing during their hospital stay.^21^ In all models, patient case-mix adjustment was undertaken using the SHMI risk score (range 0%-100% indicating estimated risk of death), with ward included as a random effect to account for unmeasured ward-level factors. Models without staffing factors gave good concordance (C-index = 0.854). The assumption of proportional hazards was examined by graphing scaled Schoenfeld residuals and was reasonable for the staffing variables.

We first assessed the association of exposure with low staffing. Because detrimental understaffing effects may accumulate over time, we modeled days of low staffing as a cumulative time-dependent covariate. We then assessed the association of staff-mix with outcomes. Initially, we estimated models including low staffing and one staff-mix factor, comparing model fit to the model with low staffing only. Staff-mix factors that improved model fit were then included in a combined multivariable model, after which we assessed interactions between staff-mix and low staffing and nonlinear associations. We used the Akaike information criterion and the Bayesian information criterion to assess model fit, preferring models that minimized the values of both.^22^

Dataset construction and analysis was undertaken using R version 4.3.0 (R Project for Statistical Computing) with Rstudio (Posit),^23,24^ data wrangling and descriptive statistics used the packages tidyverse 2.0.0 and finalfit 1.0.6.^25,26^ Survival dataset construction and analysis used survival 3.5-5 and coxme 2.2-18.1 packages.^27,28^ Significance was considered a 2-sided *P* < .05. Data analysis was conducted from April 2022 to June 2023.

## Results

We linked data for 626 313 admissions (319 518 aged 65 years or older [51.0%]; 348 464 female [55.6%]) to 185 inpatient units across 4 trusts (Table 1). Of all admissions, 502 717 (80.3%) were emergencies and 412 403 (65.8%) were to medical specialties. See eTable 2 in Supplement 1 for diagnostic groups. The median (IQR) hospital stay was 3.63 (1.77-8.28) days. Most admissions had at least 1 comorbidity with 279 415 (44.6%) having a Charlson Comorbidity Index score greater than 5. Mortality within 30 days of admission was 5.1% (31 885 admissions).

**Table 1.** Adult Inpatient Admissions During the Study Period

Characteristic	Admissions, No. (%)		
	All (N = 626 313)	Alive (n = 594 428)^a^	Dead (n = 31 885)^a^
Sex			
Female	348 464 (55.6)	333 411 (56.1)	15 053 (47.2)
Male	277 849 (44.4)	261 017 (43.9)	16 832 (52.8)
Emergency admission	502 717 (80.3)	471 895 (79.4)	30 822 (96.7)
Age group, y			
15-19	13 699 (2.2)	13 665 (2.3)	34 (0.1)
20-24	25 980 (4.1)	25 927 (4.4)	53 (0.2)
25-29	33 779 (5.4)	33 702 (5.7)	77 (0.2)
30-34	36 544 (5.8)	36 413 (6.1)	131 (0.4)
35-39	29 740 (4.7)	29 543 (5.0)	197 (0.6)
40-44	23 420 (3.7)	23 131 (3.9)	289 (0.9)
45-49	28 092 (4.5)	27 589 (4.6)	503 (1.6)
50-54	34 560 (5.5)	33 756 (5.7)	804 (2.5)
55-59	38 843 (6.2)	37 595 (6.3)	1248 (3.9)
60-64	42 138 (6.7)	40 401 (6.8)	1737 (5.4)
65-69^b^	49 617 (7.9)	47 071 (7.9)	2546 (8.0)
70-74	60 212 (9.6)	56 532 (9.5)	3680 (11.5)
75-79	59 095 (9.4)	54 974 (9.2)	4121 (12.9)
80-84	60 436 (9.6)	55 156 (9.3)	5280 (16.6)
85-89	51 579 (8.2)	45 983 (7.7)	5596 (17.6)
90-120	38 579 (6.2)	32 990 (5.5)	5589 (17.5)
Charlson Comorbidity Index score			
0	245 515 (39.2)	242 986 (40.9)	2529 (7.9)
1-5	100 037 (16.0)	97 842 (16.5)	2195 (6.9)
>5	279 415 (44.6)	252 275 (42.4)	27 140 (85.1)
Length of stay, d			
Mean (SD)	7.85 (13.88)	7.79 (14.15)	9.0 (7.00)
Median (IQR)	3.63 (1.77-8.28)	3.46 (1.72-8.00)	7.02 (3.25-13.02)

^a^ Within 30 days of admission.

^b^ Median age group.

Mean (SD) staffing over the first 5 days was 5.29 (4.22) RN HPPD and 2.93 (1.37) NS HPPD (Table 2). Low staffing (below the ward mean) occurred on 1 116 749 of 2 468 860 patient days (45.2%). The mean (SD) RN proportion was 61.50% (0.12%). Mean (SD) RN hours included 25.44% (0.18%) from senior staff (band 6 and above), 5.20% (0.10%) from temporary bank staff, and 4.84% (0.10%) from temporary agency staff. Of NS hours, a mean (SD) 1.97% (0.06%) were provided by senior staff (band 4). Nearly 14% of NS hours were provided by temporary staff, mostly bank (mean [SD], 12.28% [0.19%]).

**Table 2.** Patient Day Staffing Characteristics for All Days and for Days of Low Staffing

Staffing type	All days (N = 626 313)		Days of low staffing (N = 283 302)^a^	
	Mean (SD)	Median	Mean (SD)	Median
RN staffing				
Hours per patient day	5.29 (4.22)	4.17	4.67 (3.88)	3.57
No. of RN, %	61.48 (0.12)	61.28	60.05 (0.13)	59.81
Senior registered nurse staff hours (band 6+), %	25.44 (0.18)	21.43	26.33 (0.20)	22.12
Temporary bank hours, %	5.20 (0.10)	0.00	4.84 (0.10)	0.00
Temporary agency hours, %	4.88 (0.10)	0.00	4.08 (0.10)	0.00
Support staffing				
Hours per patient day	2.93 (1.37)	2.74	2.72 (1.19)	2.58
Senior nursing support (band 4) hours, %	1.97 (0.06)	0.00	2.19 (0.07)	0.00
Temporary bank hours, %	12.28 (0.19)	0.00	11.15 (0.19)	0.00
Temporary agency hours, %	1.50 (0.06)	0.00	1.15 (0.05)	0.00

Abbreviation: RN, registered nurse.

^a^ The total number of patient-days below the mean was 1 116 749 of 2 468 860 patient-days (45.2%).

Of the 514 899 patients exposed to days of low RN staffing, 27 397 (5.3%) died, whereas 4488 of 111 414 (4.0%) who were not exposed died. Results were similar for patients exposed to days of low NS staffing. In the multivariable model, each day of low RN staffing was associated with a 7.9% increase in risk of death (adjusted hazard ratio [aHR], 1.08; 95% CI, 1.07-1.09; *P* < .001) and each day of low NS staffing was associated with a 7.2% increase in risk (aHR, 1.07; 95% CI, 1.06-1.08; *P* < .001) (Table 3).

**Table 3.** Understaffing, Staff Mix, and Mortality (Linear Effects)^a^^,^^b^

Outcome	Model 1: low staffing only		Model 2: low staffing and staff mix	
	Mortality, adjusted HR (95% CI)^c^	*P* value	Mortality, adjusted HR (95% CI)^c^	*P* value
SHMI (mortality risk)	1.06 (1.06-1.06)	<.001	1.06 (1.06-1.06)	<.001
Days of understaffing				
RN	1.08 (1.07-1.09)	<.001	1.08 (1.07-1.09)	<.001
NS	1.07 (1.06-1.08)	<.001	1.08 (1.07-1.09)	<.001
Proportion of senior staff				
RN	NA	NA	0.99 (0.98-1.00)	.20
NS	NA	NA	0.98 (0.96-1.01)	.17
Proportion of bank staff				
RN	NA	NA	1.02 (1.01-1.04)	.005
NS	NA	NA	1.02 (1.01-1.03)	<.001
Proportion of agency staff				
RN	NA	NA	1.02 (1.01-1.04)	<.001
NS	NA	NA	1.04 (1.02-1.06)	<.001
Ward random effect, SD	1.205	NA	1.206	NA

Abbreviations: HR, hazard ratio; NA, not applicable; NS, nursing support; RN, registered nurse; SHMI, summary hospital-level mortality indicator.

^a^ Akaike information criterion: 788 843 (model 1) and 788 748 (model 2).

^b^ Bayesian information criterion: 790 453 (model 1) and 790 409 (model 2).

^c^ Adjusted for mortality risk and ward.

Results were similar when low staffing was defined at different thresholds below the mean, while the estimated adverse effects of low RN staffing increased with thresholds above the mean (eFigure 1 in Supplement 1). Models using the first 3 and 10 days of staffing gave similar results to those using 5 days (eTable 3 in Supplement 1). Models with ward as a fixed effect gave similar estimates for staffing effects, as did models including weekend admission as a factor.

Adding single staff-mix factors to the low staffing model improved model fit, except for the proportion of RNs. A higher proportion of senior RNs was associated with a lower risk of death (aHR, 0.99; 95% CI, 0.97-1.00; *P* = .005) whereas higher proportions of temporary staff were associated with a higher risk of death (eTable 4 in Supplement 1). Staff-mix factors leading to improved model fit were then included in a combined multivariable model (Table 3).

Every 10% increase in the proportion of temporary RNs (bank and agency) was associated with a 2.3% increase in risk of death for both bank temporary staff (aHR, 1.02; 95% CI, 1.01-1.04; *P* = .005) and agency temporary staff (aHR, 1.02; 95% CI, 1.01-1.04; *P* < .001). Increases in bank assistants had a similar association (aHR, 1.02; 95% CI, 1.01-1.03; *P* < .001), but every 10% increase in the proportion of agency assistants was associated with a 4% increase in risk (aHR, 1.04; 95% CI, 1.02-1.06). Small reductions in risk were observed with increased proportions of senior RN and NS staff, but they were not statistically significant. If the senior RN was defined as band 7 and above, the conclusion was unaltered.

We tested for nonlinear associations, adding polynomial terms for staffing variables. The marginal effect of understaffing increased when patients were exposed to more days of understaffing while the marginal effect of a higher proportion of bank assistants and senior NS staff reduced as levels approached 20% (eFigure 2 in Supplement 1); however, model fit was not improved (increased bayesian information criterion) (eTable 5 in Supplement 1). We tested for interactions between low staffing and the staff-mix variables of grade-mix and temporary staffing. While some interactions were statistically significant, plots suggested trivial effects (see eFigure 3 in Supplement 1 for examples) and overall model fit was worse.

We estimated the net effects of rectifying low staffing using temporary staff. We used hazard ratio estimates from Table 3 (model 2) and applied them to the mean staffing levels and shortfalls in Table 2, assuming that observed shortfalls were removed by temporary staff, thus increasing the proportion of temporary staff (eAppendix and eTable 6 in Supplement 1). If using temporary staff to avoid shortfalls, the resulting increase in temporary staff partially offsets the benefit from avoiding low staffing, leading to an estimated net reduction in the risk of death of 4.1% for low staffing averted using temporary RNs (bank or agency) or bank NS, compared with a decrease of 7.7% associated with rectifying low staffing with permanent RNs. When agency NS staff were used to address shortfalls of support staff, the reduction in risk of death was 1.2%.

## Discussion

In this cohort study, when patients were exposed to days of low nurse staffing, the risk of death was substantially increased. The mix of staff was associated with variation in the risk of death, but there was no evidence that more senior staff or temporary staff could fully compensate for the effect of low staffing. There was some evidence that having more senior staff within the RN and NS staff groups was associated with reduced risk of death, but results were inconsistent. Higher proportions of temporary staff were associated with an increased risk of death. Agency NS staff had a larger adverse association than those employed through the hospital bank. To our knowledge, this is the first study to explore the offsets between low staffing and temporary staff use. While the benefits of avoiding low staffing were greater than the harms associated with temporary staff, net benefits from more staff were diminished, and, in the case of agency-employed NS staff, negligible.

Our findings on the adverse outcomes of low RN staffing are consistent with a growing body of evidence, which includes an increasing number of longitudinal studies examining individual patient exposures to low staffing levels.^3^ The adverse association of low RN staffing was greater than that of NS staffing, but low NS staffing was still associated with increased risk of death. We found no evidence that the proportion of RNs was associated with increased mortality. This finding seems to go against a considerable body of prior research, which has mostly found that a greater presence of RNs in the skill-mix is associated with a reduction in adverse events.^6,7^ Other studies using methods similar to those of this study have concluded that having adequate support staff is important for patient safety,^29^ but the preponderance of evidence, including some pointing to adverse effects arising from both high and low support staffing levels^30^ should caution against a simplistic interpretation of this evidence as supporting substitution. Rather, we interpret this finding as indicating that adequate staffing of the nursing team, including support staff, is important for maintaining patient safety.

Previous studies support a beneficial effect from the use of more senior staff within the nursing team although these studies have not considered the mix of the support team.^14^ Our finding is consistent with some benefits from the relatively new RN associate role, which will increase the proportion of senior support staff. The possibility of a nonlinear association in our findings suggests a diminishing return and even adverse effects at higher levels, but conclusions must be tentative because model fit was not improved. Evidence that more senior RNs reduced the risk of death was mixed in that the effect was reduced and not statistically significant in models that included all staff-mix factors.

Previous research on the use of temporary staff has led to mixed conclusions. Aiken and colleagues^12,13^ concluded that an apparent association of high use of temporary staffing and adverse staff with patient outcomes could be attributed to poor work environments and lower staffing levels in hospitals using more temporary staff. Hurst et al^31^ concluded that wards using temporary staff were more expensive to run but could deliver similar quality of care. On the other hand, a UK study^11^ found that days with high use of temporary staffing were associated with increased risk of death independently of low staffing. Our study also found that higher proportions of temporary staff were associated with increased risk, irrespective of low staffing, with no important interaction between the two. We were able to assess the possible impact of trade-offs between risks from low staffing and risks from using temporary staff. The harm associated with low staffing was greater than that associated with using temporary staff to rectify any shortfall. While this finding is reassuring, use of temporary staff to rectify shortfalls is unlikely to be cost-effective because outcomes are worse with likely higher costs.^32^ We did not observe any difference for temporary RNs employed through the hospital bank compared with an agency, but agency support staff had little, if any, contribution to maintaining patient safety.

### Strengths and Limitations

Our research has several strengths. We were able to use objective longitudinal data to explore the association of variation in staffing over time with the risk of death. We included many inpatient units, and the trusts were diverse. However, this study also has limitations. The study was observational, so cause cannot be established. We used mean observed staffing as a reference point for expected staffing to account for different staffing requirements in different wards; however, mean staffing is influenced by factors other than patient need. We considered the grade of staff within RN and NS staff groups as a proxy for skills and experience. Although the bands are based on assessments of the knowledge skills and judgement required to perform a role, we had no direct measure nor could we assess variation within grades. We focused on only a single outcome, but low staffing is known to have adverse effects on a range of outcomes for patients, quality of care, and staff. The effects of staff-mix on these outcomes may differ. Further qualitative and quantitative research to understand the work of temporary staff would be of value. We used model coefficients to estimate the trade-offs between risks from low staffing and risks from using temporary staff, but this assumes the associations were causal and the estimates were unbiased.

## Conclusions

In this cohort study, low levels of nurse staffing in hospital wards were associated with an increased risk of death. Having sufficient seniority and experience within the nursing team was important, but it did not provide a strategy to mitigate the adverse effects of staff shortfalls. While the benefits of avoiding low staffing were greater than the harms associated with using temporary staff, particularly for RNs, risk remained elevated if temporary staff were used to fill staffing shortages. While our findings show the importance of considering the mix of staff as well as absolute numbers, the largest effect sizes observed were those associated with low staffing. This finding challenges the assumption that temporary staff are a cost-effective long-term solution to maintaining patient safety.

## Notes

Supplement 1. **eTable 1.** Contextual Data for Participating Trusts (2019) **eTable 2.** Diagnostic Groups in Sample (Main Diagnostic Group and Comorbidities) **eFigure 1.** Low Staffing Effects Using Alternate Thresholds for Low Staffing **eTable 3.** Sensitivity Analysis: Different Exposure Windows for Low Staffing **eTable 4.** Effects of Low Staffing and Staff-Mix on Mortality - Survival Models With Single Staff-Mix Factors **eFigure 2**. Nonlinear Relationship: Support Staff-Mix and Hazard of Death **eTable 5.** Combined Model for Associations Between Staff-Mix and Mortality (Linear and Nonlinear) **eFigure 3.** Interaction Between Low Staffing and Staff-Mix **eAppendix.** Estimating the Effects of Eliminating Low Staffing With Temporary Staff **eTable 6.** Data Used to Estimate Net Effects of Averting Low Staffing Using Temporary Staff

Supplement 2.
Nonauthor Collaborators

Supplement 3.
Data Sharing Statement
