# Supplementary material for: Nursing Team Composition and Mortality Following Acute Hospital Admission
Source: JAMA Netw Open. 2024 Aug 19;7(8):e2428769. doi: 10.1001/jamanetworkopen.2024.28769 (PMC11333978; doi:10.1001/jamanetworkopen.2024.28769)
Supplement: Supplement 1. — eTable 1. Contextual Data for Participating Trusts (2019) eTable 2. Diagnostic Groups in Sample (Main Diagnostic Group and Comorbidities) eFigure 1. Low Staffing Effects Using Alternate Thresholds for Low Staffing eTable 3. Sensitivity Analysis: Different Exposure Windows for Low Staffing eTable 4. Effects of Low Staffing and Staff-Mix on Mortality - Survival Models With Single Staff-Mix Factors eFigure 2. Nonlinear Relationship: Support Staff-Mix and Hazard of Death eTable 5. Combined Model for Associations Between Staff-Mix and Mortality (Linear and Nonlinear) eFigure 3. Interaction Between Low Staffing and Staff-Mix eAppendix. Estimating the Effects of Eliminating Low Staffing With Temporary Staff eTable 6. Data Used to Estimate Net Effects of Averting Low Staffing Using Temporary Staff [file jamanetwopen-e2428769-s001.pdf]

Supplemental Online Content

Griffiths P, Saville C, Ball J, et al; Workforce Health Outcomes Study Group. Beyond numbers—nursing team composition and mortality following acute hospital admission. *JAMA Netw. Open*. 2024;7(8):e2428769.
doi:10.1001/jamanetworkopen.2024.28769

eTable 1. Contextual Data for Participating Trusts (2019)
eTable 2. Diagnostic Groups in Sample (Main Diagnostic Group and Comorbidities)
eFigure 1. Low Staffing Effects Using Alternate Thresholds for Low Staffing
eTable 3. Sensitivity Analysis: Different Exposure Windows for Low Staffing
eTable 4. Effects of Low Staffing and Staff-Mix on Mortality - Survival Models With Single Staff-Mix Factors
eFigure 2. Nonlinear Relationship: Support Staff-Mix and Hazard of Death
eTable 5. Combined Model for Associations Between Staff-Mix and Mortality (Linear and Nonlinear)
eFigure 3. Interaction Between Low Staffing and Staff-Mix
eAppendix. Estimating the Effects of Eliminating Low Staffing With Temporary Staff
eTable 6. Data Used to Estimate Net Effects of Averting Low Staffing Using Temporary Staff

This supplemental material has been provided by the authors to give readers additional information about their work

© 2024 Griffiths P et al. *JAMA Network Open.*

eTable 1. Contextual Data for Participating Trusts (2019)[a]

	Teaching	Region	Beds (occupancy)[1]	Admissions[2]	RN / occupied bed[3]	Medics per occupied bed	AHP	Mortality rate	SHMI	Staff satisfaction[4]	Patient satisfaction[5]
Trust 1	Y	Mid	1746 (87%)	139775	2.02	1.11	0.44	3.2%	103%	56%	7.91
Trust 2	N	SE	1056(96%)	83595	1.67	0.88	0.31	3.8%	105%	64%	8.01
Trust 3	N	SW	523 (94%)	38355	1.38	0.72	0.34	2.7%	101%	68%	8.33
Trust 4	Y	Lon	993 (79%)	117990	2.74	1.71	0.68	1.1%	69%	63%	8.34
National median			753 (90%)[6]	66440	1.64	0.87	0.44	3.3%	101%	63%	7.91

1. Source: Average daily number of available and occupied general acute beds open overnight April 2018 to March 2019 NHS England: SDCS data collection - KH03
2. Spells contributing to SHMI March 2019 (April 2018-March 2019)
3. Staff per bed based on annual workforce reports 2018-19 using groups defined in section 3.1 above
4. Staff satisfaction survey 2019 – Percentage of staff selecting Agree or Strongly Agree for q21c - I would recommend my organisation as a place to work. Benchmark group is acute and acute & community.
5. 2019 Patient survey – average score overall rating / 10
6. Median calculated from all Trusts classified as acute (small, medium, large) or acute teaching in NHS benchmark groups

[a]The participating hospital Trusts were diverse in many respects including registered nurse, medical and allied health professional staffing levels, size, teaching status and region (see eTable 1). In three Trusts acute inpatient services were largely provided from a single site. The largest Trust included 100 units (wards) while the smallest included 29. Trust 1 was in an urban area with a relatively high proportion of people born outside the UK (25% vs 17% national average) with 15% identifying as Asian ethnicity (the largest non-white ethnic group) in the 2021 census. A higher-than-average proportion was economically inactive (46% vs 39%), and a lower-than-average proportion were in higher managerial, administrative, and professional occupations (9% vs 13%). Trust 2 was also in an urban area with a proportion born outside the UK of about the national average (17%) with 7% identifying as Asian ethnicity (the largest non-white ethnic group). Slightly fewer people than the national average were economically inactive (38% vs 39%) but a lower-than-average proportion were in higher managerial, administrative and professional occupations (10% vs 13%). Trust 3 was in a small town in a primarily affluent rural area. There was a lower-than-average proportion of people born outside the UK (11% vs 17%) and the largest non-white ethnic group (Asian) was only 3% of the population. In contrast with the previous two Trusts the age profile included higher proportions of older people, a lower-than-average proportion were economically inactive (37.5% vs 39%), and a higher-than-average proportion were in higher managerial, administrative, and professional occupations (15% vs 13%). Trust 4 was in greater London, with the local area having an ethnically diverse population with a high proportion born outside the UK (45% vs 17%) and 18% identifying as of an Asian ethnic background. A lower-than-average proportion were economically inactive (37% vs 39%) and a much higher than average proportion were in higher managerial, administrative, and professional occupations (24% vs 13%) although the area also included a higher-than-average proportion of those who had never worked or were long-term unemployed (10% vs 8.5%). The patient population was diverse with 138 different diagnostic groups represented (see eTable 2). One-third (213868) of admissions were classified as surgical with the remainder classified as medical (including all non-surgical specialities)

© 2024 Griffiths P et al. *JAMA Network Open.*

eTable 2. Diagnostic Groups in Sample (Main Diagnostic Group and Comorbidities)

**Primary diagnostic group for admission**	
Diagnostic group*	N (%)
Abdominal hernia	5405 (0.9)
Abdominal pain	8685 (1.4)
Acute and unspecified renal failure	5569 (0.9)
Acute bronchitis	7614 (1.2)
Acute cerebrovascular disease	15529 (2.5)
Acute myocardial infarction	11463 (1.8)
Allergic reactions, aftercare & screening, R codes	5846 (0.9)
Anaemia	3701 (0.6)
Aortic & peripheral arterial embolism or thrombosis	466 (0.1)
Aortic; peripheral; and visceral artery aneurysms	1438 (0.2)
Appendiceal conditions, Peritonitis & intestinal abscess	4517 (0.7)
Aspiration pneumonitis; food/vomitus	2092 (0.3)
Asthma	4095 (0.7)
Back problems, osteoporosis	8296 (1.3)
Bacterial infection; unspecified site	593 (0.1)
Benign neoplasm	5227 (0.8)
Biliary tract disease	10371 (1.7)
Burns	1225 (0.2)
Cancer of bladder	2856 (0.5)
Cancer of bone, thyroid and malignant neoplasm	2727 (0.4)
Cancer of brain and nervous system	1659 (0.3)
Cancer of breast	3388 (0.5)
Cancer of bronchus; lung	3491 (0.6)
Cancer of colon	2374 (0.4)
Cancer of female genital organs	767 (0.1)
Cancer of head and neck	2374 (0.4)
Cancer of liver and intrahepatic bile duct	950 (0.2)
Cancer of male reproductive organs	2917 (0.5)
Cancer of oesophagus	1405 (0.2)
Cancer of other GI organs; peritoneum	554 (0.1)
Cancer of ovary	734 (0.1)
Cancer of pancreas	822 (0.1)
Cancer of rectum and anus	1709 (0.3)
Cancer of stomach	1008 (0.2)
Cancer of urinary organs	1344 (0.2)
Cancer of uterus	798 (0.1)
Cancer; other respiratory and intrathoracic	304 (0.0)
Cardiac & circulatory congenital anomalies	277 (0.0)
Cardiac arrest and ventricular fibrillation	574 (0.1)
Cardiac dysrhythmias	8139 (1.3)
Central nervous system infections	1076 (0.2)
Coma; stupor; and brain damage	663 (0.1)
Complication of device; implant; or graft	11435 (1.8)
Complication of surgical procedures or medical care	9218 (1.5)
Conduction disorders	1608 (0.3)
Congenital anomalies	1288 (0.2)
Congestive heart failure; nonhypertensive	8648 (1.4)
COPD & bronchiectasis	14797 (2.4)
Coronary atherosclerosis and other heart disease	7319 (1.2)
Crushing injury or internal injury	3018 (0.5)

© 2024 Griffiths P et al. *JAMA Network Open.*

Cystic fibrosis, Other lower respiratory disease	3316 (0.5)
Diabetes mellitus with complications	3215 (0.5)
Diabetes mellitus without complication	1118 (0.2)
Digestive, anal and rectal conditions	4468 (0.7)
Disease of veins and lymphatics	1848 (0.3)
Diseases of kidneys and ureters, bladder and urethra	8067 (1.3)
Diseases of white blood cells	571 (0.1)
Disorders of stomach and duodenum	1610 (0.3)
Ear and sense organ disorders (excluding TB/STD)	10372 (1.7)
Epilepsy; convulsions	4497 (0.7)
Esophageal disorders	2188 (0.4)
Female genital/reproductive disorders	5737 (0.9)
Fever of unknown origin	1248 (0.2)
Fluid and electrolyte disorders	4636 (0.7)
Fracture of lower limb	6304 (1.0)
Fracture of neck of femur (hip)	8470 (1.4)
Fracture of upper limb	4347 (0.7)
Gastroduodenal ulcer (except hemorrhage)	410 (0.1)
Gastrointestinal hemorrhage	6457 (1.0)
Genitourinary symptoms and ill-defined conditions	4702 (0.8)
Heart valve disorders	2271 (0.4)
Hematologic conditions	1190 (0.2)
Hereditary & degenerative nervous system conditions	1799 (0.3)
HIV infection	94 (0.0)
Hodgkin's disease	230 (0.0)
Hypertension	768 (0.1)
Infective arthritis and osteomyelitis (excluding TB/STD)	1406 (0.2)
Intestinal infection	8143 (1.3)
Intestinal obstruction without hernia	4518 (0.7)
Intracranial injury	3872 (0.6)
Joint disorders, fractures & sprains	10749 (1.7)
Leukemias	2088 (0.3)
Liver disease; alcohol-related	1658 (0.3)
Lung disease due to external agents	151 (0.0)
Lymphadenitis, Gangrene	411 (0.1)
Malaise and fatigue	654 (0.1)
Male genital disorders	3110 (0.5)
Melanomas, other cancer of skin	998 (0.2)
Multiple myeloma	1260 (0.2)
Mycoses	288 (0.0)
Nausea and vomiting	2610 (0.4)
Neoplasms (unspecified), Nonmalignant breast conditions	1470 (0.2)
Nephritis; nephrosis; renal sclerosis, Chronic renal failure	5962 (1.0)
Non-HIV related infections	3009 (0.5)
Non-Hodgkin's lymphoma	2516 (0.4)
Noninfectious gastroenteritis	1159 (0.2)
Non-organic mental disorders, anxiety	4120 (0.7)
Nonspecific chest pain	6428 (1.0)
Nutritional, endocrine and metabolic disorders	2923 (0.5)
Open wounds of extremities	2683 (0.4)
Open wounds of head; neck; and trunk	2497 (0.4)
Organic mental disorders	4986 (0.8)
Other and ill-defined heart disease	225 (0.0)
Other cancer (primary)	272 (0.0)
Other cerebrovascular disease	2883 (0.5)

© 2024 Griffiths P et al. *JAMA Network Open.*

Other circulatory disease	3644 (0.6)
Other connective tissue disease	12035 (1.9)
Other gastrointestinal disorders	6132 (1.0)
Other injuries & conditions due to external causes	1039 (0.2)
Other liver diseases	2246 (0.4)
Other nervous system disorders	6761 (1.1)
Other non-traumatic joint disorders	3465 (0.6)
Other psychoses	1427 (0.2)
Pancreatic disorders (not diabetes)	4323 (0.7)
Paralysis, Late effects of cerebrovascular disease	867 (0.1)
Parkinson's disease	733 (0.1)
Pathological fracture	1138 (0.2)
Peri / endo / myocarditis, cardiomyopathy	1429 (0.2)
Peripheral and visceral atherosclerosis	2801 (0.4)
Pleurisy; pneumothorax; pulmonary collapse	3778 (0.6)
Pneumonia (excluding TB/STD)	27701 (4.4)
Poisoning	6203 (1.0)
Pregnancy related conditions	59069 (9.5)
Psychotic disorders, other mental conditions	349 (0.1)
Pulmonary heart disease	2796 (0.4)
Regional enteritis and ulcerative colitis	2307 (0.4)
Respiratory failure; insufficiency; arrest (adult)	1045 (0.2)
Rheumatoid arthritis related diseases, acquired deformities, bone disease	21687 (3.5)
Secondary malignancies	6964 (1.1)
Septicaemia (except in labour), Shock	17980 (2.9)
Skin and subcutaneous tissue infections	8672 (1.4)
Skin disorders	2833 (0.5)
Superficial injury; contusion	4272 (0.7)
Syncope	4858 (0.8)
Thyroid disorders, Other endocrine disorders	3017 (0.5)
Tuberculosis	172 (0.0)
Upper respiratory disease, Diseases of mouth (non dental)	15137 (2.4)
Urinary tract infections	12611 (2.0)
**All**	624846 (100.0)
**Comorbidities****	
Acute myocardial infarction	1211 (0.5)
Cancer	16950 (6.5)
Cerebral vascular accident	482 (0.2)
Congestive heart failure	26214 (10.1)
Connective tissue disorder	59652 (23.0)
Dementia	12478 (4.8)
Diabetes	4458 (1.7)
Diabetes complications	10750 (4.1)
HIV	9536 (3.7)
Liver disease	9778 (3.8)
Metastatic cancer	6153 (2.4)
Paraplegia	15007 (5.8)
Peptic ulcer	38750 (14.9)
Peripheral vascular disease	7621 (2.9)
Pulmonary disease	14819 (5.7)
Renal disease	19258 (7.4)
Severe liver disease	6737 (2.6)

© 2024 Griffiths P et al. *JAMA Network Open.*

*Diagnostic groups used in the Standardised Hospital Mortality Indicator model – see NHS Digital. Summary Hospital-level Mortality Indicator (SHMI) - Deaths associated with hospitalisation, England. August 15, 2023. Accessed August 15, 2023. https://digital.nhs.uk/data-and-information/publications/clinical-indicators/shmi/current for full definitions and ICD-10 codes included in each group

** Charlson comorbidities (each admission could have more than one)

© 2024 Griffiths P et al. *JAMA Network Open.*

eFigure 1. Low Staffing Effects Using Alternate Thresholds for Low Staffing

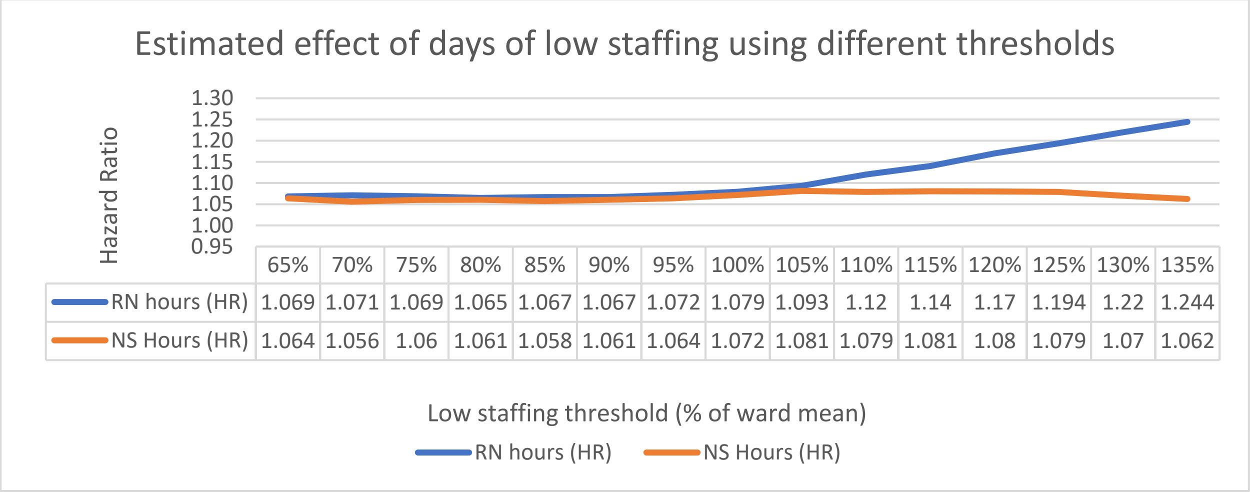

	65%	70%	75%	80%	85%	90%	95%	100%	105%	110%	115%	120%	125%	130%	135%
RN hours (HR)	1.069	1.071	1.069	1.065	1.067	1.067	1.072	1.079	1.093	1.12	1.14	1.17	1.194	1.22	1.244
NS Hours (HR)	1.064	1.056	1.06	1.061	1.058	1.061	1.064	1.072	1.081	1.079	1.081	1.08	1.079	1.07	1.062

© 2024 Griffiths P et al. *JAMA Network Open.*

eTable 3. Sensitivity Analysis: Different Exposure Windows for Low Staffing

**5-day exposure window**	**Hazard Ratio (HR)**	**LCL**	**UCL**	**P**
SHMI	1.063	1.062	1.064	<0.001
Days of low RN HPPD	1.079	1.070	1.089	<0.001
Days of low NS HPPD	1.072	1.062	1.081	<0.001
Ward random effect (sd)	1.205	AIC	BIC	
Model fit		788843.	790453	
**3-day exposure window**	**HR**	**LCL**	**UCL**	**P**
SHMI	1.065	1.064	1.066	<0.001
Days of low RN HPPD	1.068	1.054	1.083	<0.001
Days of low NS HPPD	1.064	1.050	1.079	<0.001
Ward random effect (sd)	1.216	AIC	BIC	
Model fit		777384	778949	
**10-day exposure window**	**HR**	**LCL**	**UCL**	**P**
SHMI	1.061	1.060	1.061	<0.001
Days of low RN HPPD	1.080	1.074	1.086	<0.001
Days of low NS HPPD	1.069	1.063	1.075	<0.001
Ward random effect (sd)	1.228	AIC	BIC	
Model fit		792880.9	794512.3	

© 2024 Griffiths P et al. *JAMA Network Open.*

eTable 4. Effects of Low Staffing and Staff-Mix on Mortality - Survival Models With Single Staff-Mix Factors

**Low staffing (only) by staff group**	**Hazard Ratio (HR)**	**LCL**	**UCL**	**P**
SHMI	1.063	1.062	1.064	<0.001
Days of low RN HPPD	1.079	1.070	1.089	<0.001
Days of low NS HPPD	1.072	1.062	1.081	<0.001
Ward random effect (sd)	1.205	AIC	BIC	
Model fit		788843	790453	
**RN skill Mix**	**HR**	**LCL**	**UCL**	**P**
SHMI	1.063	1.062	1.064	<0.001
Days of low RN HPPD	1.083	1.072	1.093	<0.001
Days of low NS HPPD	1.067	1.057	1.078	<0.001
Proportion of RNs (10%)	1.013	0.997	1.030	0.100
Ward random effect (sd)	1.206	AIC	BIC	
Model fit		788842	790461	
**Grade mix**	**HR**	**LCL**	**UCL**	**P**
SHMI	1.063	1.062	1.064	<0.001
Days of low RN HPPD	1.082	1.072	1.092	<0.001
Days of low NS HPPD	1.070	1.061	1.080	<0.001
Proportion of senior RNs (10%)	0.985	0.974	0.995	0.005
Proportion of senior NAs(10%)	0.979	0.955	1.003	0.087
Ward random effect (sd)	1.195	AIC	BIC	
Model fit		788814	790439	
**Temporary staffing (bank)**	**HR**	**LCL**	**UCL**	**P**
SHMI	1.063	1.062	1.064	<0.001
Days of low RN HPPD	1.081	1.071	1.091	<0.001
Days of low NS HPPD	1.079	1.069	1.089	<0.001
Proportion of bank RNs (10%)	1.024	1.008	1.040	0.003
Proportion of bank NS (10%)	1.023	1.015	1.032	<0.001
Ward random effect (sd)	1.211	**AIC**	**BIC**	
Model fit		788796	790425	
**Temporary staffing (agency)**	**HR**	**LCL**	**UCL**	**P**
SHMI	1.063	1.062	1.064	<0.001
Days of low RN HPPD	1.081	1.071	1.090	<0.001
Days of low NS HPPD	1.077	1.067	1.086	<0.001
Proportion of agency RNs (10%)	1.032	1.019	1.044	<0.001
Proportion of agency NS (10%)	1.043	1.023	1.062	<0.001
Ward random effect (sd)	1.201	**AIC**	**BIC**	
Model fit		788808	790434	

HR - Hazard ratio, NS – Nursing support staff, RN registered nurse, HPPD hours per patient day, LCL – lower 95% confidence limit, UCL upper 95% confidence limit AIC Akaike Information Criterion BIC Bayesian Information Criterion.

© 2024 Griffiths P et al. *JAMA Network Open.*

eFigure 2. Nonlinear Relationship: Support Staff-Mix and Hazard of Death

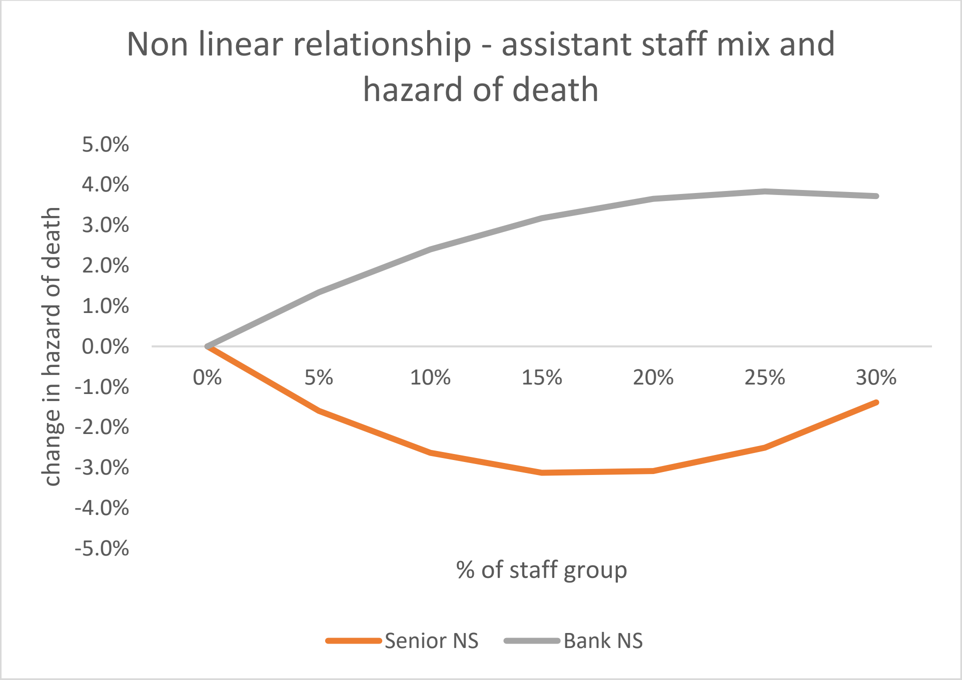

© 2024 Griffiths P et al. *JAMA Network Open.*

eTable 5. Combined Model for Associations Between Staff-Mix and Mortality (Linear and Nonlinear)

	Linear terms only model				Model with non-linear (^2) terms			
	**HR**	**LCL**	**UCL**	**P**	**HR**	**LCL**	**UCL**	**P**
SHMI	1.063	1.062	1.064	**<0.001**	1.063	1.062	1.063	**<0.001**
Days of RN understaffing	1.083	1.074	1.093	**<0.001**	1.067	1.054	1.080	**<0.001**
RN understaffing^2					1.012	1.006	1.018	**<0.001**
Days of NS understaffing	1.081	1.071	1.091	**<0.001**	1.067	1.054	1.080	**<0.001**
NS understaffing^2					1.009	1.003	1.015	**0.002**
Proportion of senior RNs	0.993	0.982	1.004	0.201	0.996	0.985	1.008	0.525
senior RNs^2					1.000	0.995	1.005	0.932
Proportion of senior NS	0.983	0.958	1.008	0.173	0.963	0.934	0.993	**0.015**
senior NS^2					1.011	1.003	1.019	**0.009**
Proportion of bank RNs	1.023	1.007	1.039	**0.005**	1.031	1.010	1.052	**0.004**
bank RNs^2					0.995	0.987	1.003	0.216
Proportion of bank NS	1.019	1.01	1.028	**<0.001**	1.030	1.019	1.041	**<0.001**
bank NS^2					0.994	0.991	0.997	**0.001**
Proportion of agency RNs	1.023	1.011	1.037	**<0.001**	1.020	1.003	1.037	**0.020**
agency RNs^2					1.001	0.994	1.008	0.787
Proportion of agency NS	1.040	1.021	1.06	**<0.001**	1.041	1.011	1.071	**0.006**
agency NS^2					0.999	0.990	1.007	0.762
Ward random effect (sd)	1.206				1.461			
Model Fit			AIC	BIC			AIC	BIC
			788748	790409			788709	790469

HR - Hazard ratio, NS – Nursing support staff, RN registered nurse, HPPD hours per patient day, LCL – lower 95% confidence limit, UCL upper 95% confidence limit AIC Akaike Information Criterion BIC Bayesian Information Criterion. **Bold *indicates statistically significant (p<0.05)***

© 2024 Griffiths P et al. *JAMA Network Open.*

eFigure 3. Interaction Between Low Staffing and Staff-Mix

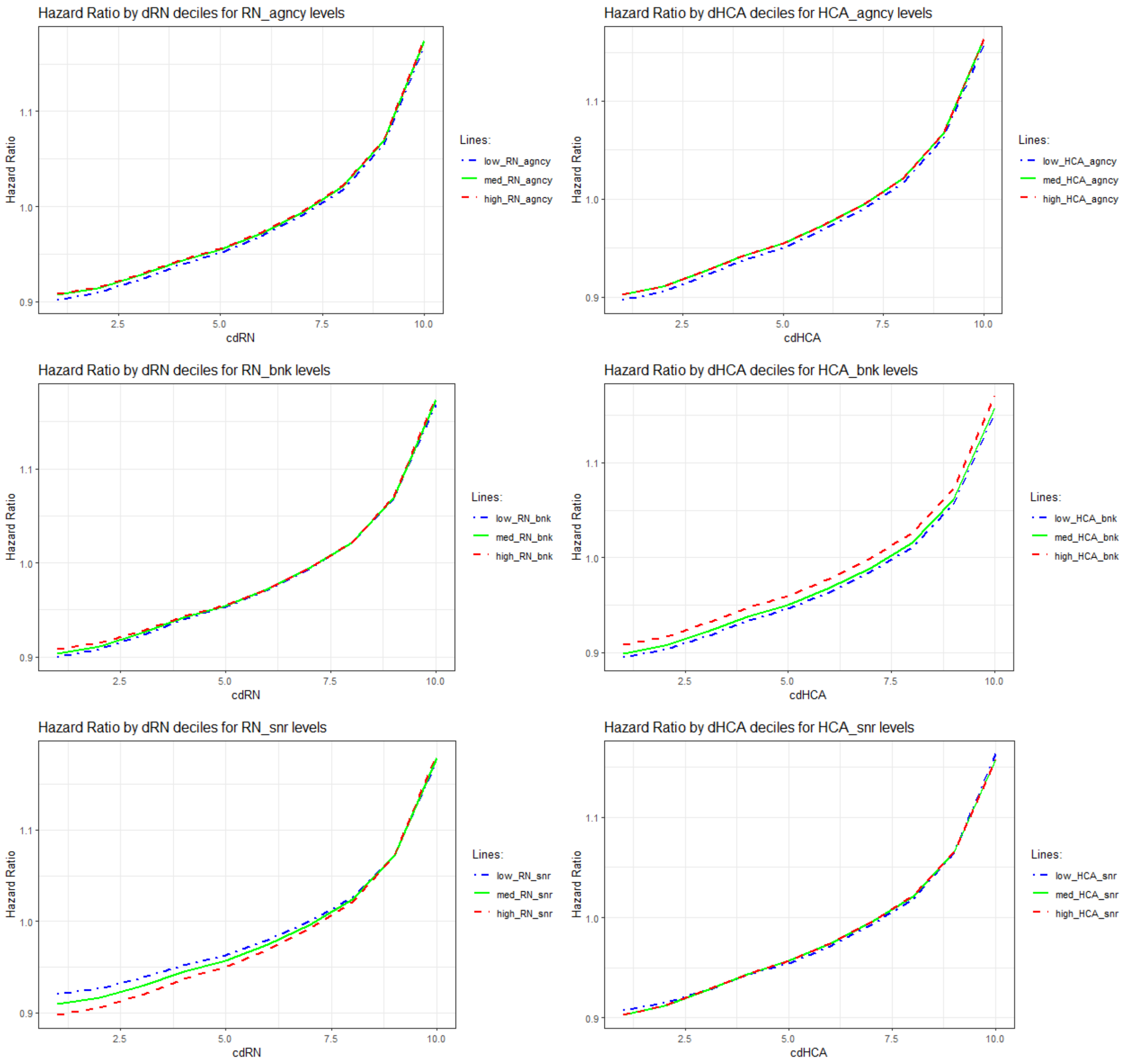

RN – registered nurse
HCA - nursing support staff (NS)
Bnk – temporary staff – bank
Snr – Senior
d – variable for proportion converted to deciles (10% intervals)

© 2024 Griffiths P et al. *JAMA Network Open.*

eAppendix. Estimating the Effects of Eliminating Low Staffing With Temporary Staff

Calculation:

- Assume Hazard ratio approximates to relative risk
- The effect of Low staffing is **HR**
- The reduction in risk of low staffing averted is therefore **1/HR=Ra**
- The effect of a 10% increase in the proportion of temporary staff is **T**
- (a or b for agency or bank)
- Using temporary staff to avert a staffing shortfall increases the % of temporary staff by **S** (shortfall as a % of mean) or $\mathbf{S_{10}}$ (expressed as 10% units).
- Net effect of averting low staffing using temporary staff is $\mathbf{Ra*T\wedge^{S10}}$

Example

Assume a permanent-only workforce as the baseline, using data from eTable 5. Low staffing is defined as below mean (expected) staffing (5.29 hppd). The mean shortfall on days of low staffing is 0.87 RN hours per patient day so avoiding this shortfall on a day when it would otherwise occur requires 0.87 RN hours (mean).

The effect of exposure to 1 day of low RN staffing is to increase the risk of death by 8.3% relative to a day without low staffing (HR 1.083).

Adding RN staff on a day that would otherwise have low staff *reduces* the risk of death. The relative change is 1/HR = (1/1.083=0.923) so avoiding a day of low staffing *reduces* the risk of death by 7.7% (relative to a day with low staffing)

If the RN staff are temporary, the proportion of temporary staff is increased compared to baseline. Using agency RNs to rectify a shortfall adds 0.87 / agency RN hours to 4.42 hours of permanent RNs. The proportion agency RNs is 0.87/5.29=0.16 (16%)

Each 10% increase of the proportion of temporary staff increases the risk of death by 2.3% (HR 1.023) so compared to 0% temporary RNs 16.4% RNs the risk of death is *increased* by 3.7% ($1.023^{\wedge 1.64}$ =1.037)

The net effect of averting low staffing using temporary RNs is thus to reduce the risk of death by 4.1% (0.923 * 1.037 = 0.959)

© 2024 Griffiths P et al. *JAMA Network Open.*

eTable 6. Data Used to Estimate Net Effects of Averting Low Staffing Using Temporary Staff

	RN	NS
Mean HPPD	5.29	2.93
Mean HPPD on days of short staffing	4.42	2.44
Mean shortfall (days of low staffing)	0.87	0.49
Shortfall as a % of mean **(S)**	16%	17%
shortfall in 10% units **(S_{10})**	1.6	1.7
Low staffing effect (HR) **(L)**	1.083	1.081
Estimated effect of low staffing averted **(La)**	0.923	0.925
Estimated reduction in risk (%)	7.7%	7.5%
Bank effects (HR per 10%) **(Tb)**	1.023	1.019
Agency effect (HR per 10%) **(Ta)**	1.023	1.04
Net effects rectify low staffing with bank (HR)	0.959	0.955
Net reduction in risk (using bank)	4.1%	4.5%
Net effects rectify low staffing with agency	0.959	0.988
Net reduction in risk (using agency)	4.1%	1.2%

© 2024 Griffiths P et al. *JAMA Network Open.*
