# Supplementary material for: Nursing Team Composition and Mortality Following Acute Hospital Admission
Source: JAMA Netw Open. 2024 Aug 19;7(8):e2428769. doi: 10.1001/jamanetworkopen.2024.28769 (PMC11333978; doi:10.1001/jamanetworkopen.2024.28769)
Supplement: Supplement 2. — Nonauthor Collaborators [file jamanetwopen-e2428769-s002.pdf]

Supplemental Online Content: Nonauthor Collaborators
*First name, last name, and suffix (if applicable) are required and will appear in PubMed.

*Group Name(s): Workforce Health Outcomes Study group							
***First Name and Middle Initial(s)**	***Last Name**	***Suffix (eg, Jr, III)**	Academic Degrees	Institution	Location (city, state/province, country)	Role or Contribution, eg, chair, principal investigator	Group (if more than 1 Group listed in the byline) and/or Subgroup (eg, Steering Committee)
Andrew	Barraclough			Nottingham University Hospitals NHS Trust	Nottingham UK	Data extraction & access	advisory group
Chris	Bojke		PhD	University of Leeds	Leeds UK	member	Steering group
Louise	Bramley			Nottingham University Hospitals NHS Trust	Nottingham UK	local principal investigator	advisory group
Greg	Bull			OceansBlue	Solihull UK	Data processing and linking	advisory group
Lara	Carmona			Royal College of Nursing	London UK	member	advisory group
Andy	Charlwood		PhD	University of Leeds	Leeds UK	Chair	Steering group
Jonathan	Drennan		PhD	University College dublin	Dublin Eire	member	Steering group
Jo	Fillingham			NHS England	London UK	member	Steering group
Liam	Flynn			Nottingham University Hospitals NHS Trust	Nottingham UK	Data extraction & access	advisory group
Fiona	Hyett			Salisbury Hospital NHS Trust	Salisbury UK	local principal investigator	advisory group
Oliver	Redfern		PhD	University of Oxford	Oxford UK	member	Steering group
Liz	Rix			Portsmouth Hospitals University NHS Trust	Portsmouth UK	local principal investigator	advisory group
Paul	Scmidt		MD	Portsmouth Hospitals University NH[illegible]	Portsmouth UK	Co-investigator	advisory group
Dave	Shields			Patient / public representative	Southampton UK	member	Steering group
Carol	Stiles			University College hospital, London	London UK	local principal investigator	advisory group
Karen	Swinson			NHS England	London UK	member	Steering group
Rachel	Taylor		PhD	University College hospital, London	London UK	data access	advisory group
Neil	Tape			University Hospital Southampton NHS Foundation Trust	Southampton UK	member	Steering group
