# Supplementary material for: Nursing Team Composition and Mortality Following Acute Hospital Admission
Source: JAMA Netw Open. 2024 Aug 19;7(8):e2428769. doi: 10.1001/jamanetworkopen.2024.28769 (PMC11333978; doi:10.1001/jamanetworkopen.2024.28769)
Supplement: Supplement 3. — Data Sharing Statement [file jamanetwopen-e2428769-s003.pdf]

Data Sharing Statement

Griffiths. Beyond Numbers—Nursing Team Composition and Mortality Following Acute Hospital Admission. *JAMA Netw Open*. Published August 19, 2024.
doi:10.1001/jamanetworkopen.2024.28769

Data
Data available: No

Additional Information
Explanation for why data not available: The nature of the data (individual patient data) and the data sharing agreement with data providers means we are unable to share data
